# Association between fibrinogen level and length of stay in patients with lower extremity atherosclerotic disease: a retrospective cohort study

**DOI:** 10.1038/s41598-023-39219-x

**Published:** 2023-07-22

**Authors:** Xue Wang, Yu Yang, Ling Yu, Chang Pang, Wei Sun, Shuang Zang, Cong Li

**Affiliations:** 1 Department of Community Nursing, School of Nursing, China Medical University, No.77 Puhe Road, Shenyang North New Area, Shenyang, 110122 Liaoning China; 2 Department of Vascular Surgery, The First Hospital of China Medical University, No.155 Nanjing Bei Street, Heping District, Shenyang, 110001 Liaoning China; 3 Phase I Clinical Trails Center, The First Hospital of China Medical University, No.155 Nanjing Bei Street, Heping District, Shenyang, 110001 Liaoning China; 4 Department of General Practice, The Second Affiliated Hospital of Shenyang Medical College, No. 20 Bei Jiu Road, Heping District, Shenyang, 110002 Liaoning China; 5 Department of Ultrasound, The Second Affiliated Hospital of Shenyang Medical College, No. 20 Bei Jiu Road, Heping District, Shenyang, 110002 Liaoning China

**Keywords:** Biomarkers, Diseases, Health care, Medical research, Risk factors

## Abstract

The level of fibrinogen in patients with lower extremity atherosclerosis (LEAD) has been widely identified as a risk factor contributing to adverse outcomes. However, some knowledge gaps remain regarding fibrinogen levels and downstream adverse outcomes, such as length of stay (LOS). We conducted this study to examine the association between fibrinogen level and LOS in LEAD patients. The retrospective cohort study included 1428 LEAD patients between January 2014 and November 2021 in China. Several generalized linear models with a negative binomial link function were used to evaluate the association between fibrinogen level and LOS. The area under the curve (AUC) was used to evaluate the predicting effect of fibrinogen level on a LOS greater than 10 days (median LOS). The median age of the patients was 70 years old, and 1153 (80.74%) were males. Fibrinogen level was positively associated with LOS (β = 1.14; 95% CI, 0.42–1.86; p = 0.002) in LEAD patients after controlling for age, gender, number of historical hospitalizations, surgical history, vascular disease history, drinking history, smoking history, insurance type, surgical approach, lesion site, weight loss, Fontaine classification, age-adjusted Charlson comorbidity index, urea, total protein, activated partial thromboplastin time, thrombin time, prothrombin time-international normalized ratio, calcium, triglyceride, albumin/globulin ratio, phosphorus, and D-dimer. The fibrinogen-added prediction model demonstrated good discrimination and calibration, with an AUC value of 0.807. Fibrinogen level was positively associated with LOS in LEAD patients. The fibrinogen level is a widely available and easy-to-measure biochemical indicator, and it could be used as a suitable indicator for the prognosis and prophylaxis of prolonged LOS in patients with LEAD during hospitalization.

## Introduction

As a manifestation of peripheral arterial disease (PAD), lower extremity atherosclerotic disease (LEAD) represents systemic atherosclerosis involving peripheral arteries^1^. LEAD and its consequences of vascular occlusion, disability, foot ulceration, and lower extremity amputation markedly decrease the quality of life and increase disability rates and financial burden for patients^2,3^. Many risk factors, including cigarette smoking, dyslipidemia, and inflammatory markers, lead to LEAD and other forms of atherosclerosis^4^. Likewise, increased vascular disease morbidity and mortality, as well as the rising burden of LEAD, are expected in the foreseeable future^5^.

Growing evidence suggests that fibrinogen level is associated with increased LEAD risk^6,7^. A pathogenic role for fibrinogen has been proposed in acute thrombotic complications and the stable form of LEAD, the extent of which correlates with the degree of functional impairment^8^. Research has shown that fibrinogen can strongly predict adverse clinical outcomes in vascular disease patients^9^. According to a study of 486 patients with PAD and cardiovascular disease comorbidities, elevated fibrinogen level is associated with poor clinical outcomes^10^. A study by Alte et al. found that PAD outpatients with high fibrinogen levels were associated with an increased risk of ischemic events and major bleeding^11^. Hou et al. observed that an elevated fibrinogen level at baseline and 90 days is associated with poor functional outcomes in ischemic stroke or transient ischemic attack patients^12^. You et al. revealed that traumatic brain injury patients with deteriorating fibrinogen values had a prolonged LOS in the intensive care unit^13^.

The LOS is an important quality measure to assess hospital efficiency and performance^14^. Reducing LOS could alleviate hospital overcrowding, save medical resources, and reduce healthcare costs. Notably, there is accumulating evidence that patients’ biochemical indicators are associated with LOS. Xue et al. reported that a high sensitivity C-reactive protein-albumin and protein-prealbumin ratio were associated with LOS in 114 patients with COVID-19^15^. The neutrophil-to-lymphocyte and monocyte-to-lymphocyte ratios were correlated with LOS in myocarditis patients^16^. A higher level of fibrinogen at presentation increased the risk of prolonged LOS in COVID-19 patients (age < 14 years)^17^. Despite previous studies showing an association between fibrinogen level and disease severity and prognosis, to the best of our knowledge, no study has examined the potential association between fibrinogen levels and LOS in patients with LEAD. Therefore, we studied the association between fibrinogen level and LOS in LEAD inpatients in China.

## Methods

### Study population

**Figure 1:**
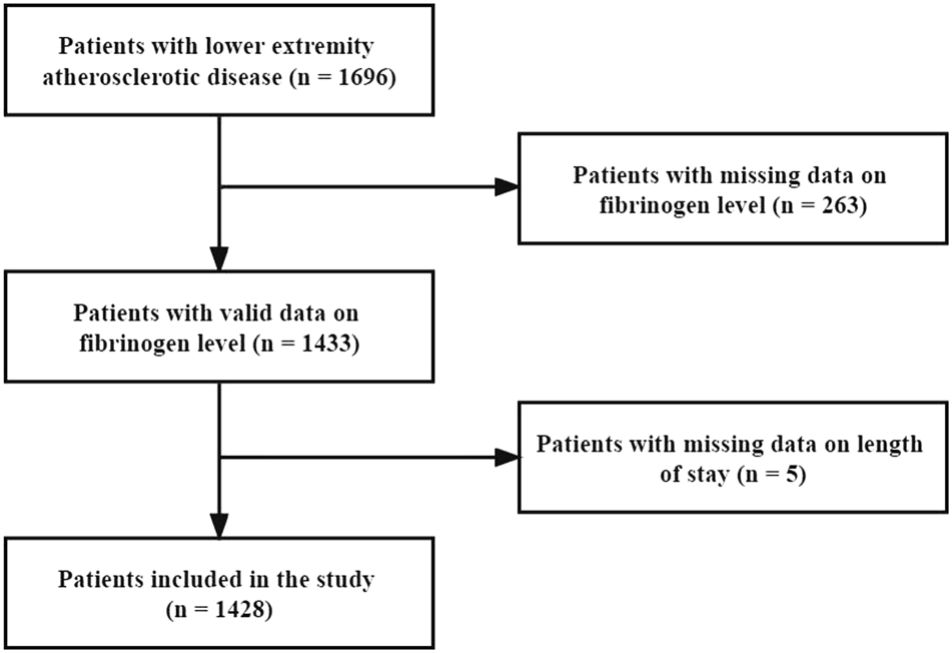
Flow chart for excluding and including patients.

From January 2014 to November 2021, a total of 1696 inpatients were admitted to the Vascular Thyroid Surgery Department in China with LEAD. LEAD diagnostic criteria were based on the Chinese version of LEAD diagnostic criteria (2007 version)^18^. The diagnosis was coded according to the International Classification of Diseases-10th (ICD-10). The discharge criteria were lifted target vessel lesions and no obvious complications. The patient’s hospitalization information data were recorded in the electronic hospital information system and included demographic data, diagnoses, medical and surgical procedures, laboratory measurements, and physiological measurements. Trained medical record abstractors extracted hospitalization data using ICD-10 codes (I70.203) for LEAD. We initially identified 1696 inpatients with LEAD. After excluding 263 patients without fibrinogen data and 5 patients with missing LOS data, the remaining 1428 patients were finally included in this study. Figure 1 shows the process of including and excluding participants in the present study.

This study was approved by the ethics committee of the First Hospital of China Medical University (ethics number: [2021] 366). As the patient information was anonymized and deidentified before analysis, the ethics committee of the First Hospital of China Medical University waived the need for informed consent. And all methods were performed following the Declaration of Helsinki.

### Exposure and outcome

The exposure variable was the fibrinogen level, which was measured after an 8-h fast on the day of admission. The blood samples for fibrinogen testing were collected in vacuum blood collection tubes, and the fibrinogen level was measured by the clotting method using an STA-R automated coagulation analyzer (Stago, France). The outcome variable was LOS, which refers to the time interval between the date of admission and the discharge^19^.

### Covariates

At the time of admission, each patient was evaluated for drinking and smoking histories, lower extremity ischemic symptoms, lesion sites, and vascular disease history. Moreover, all patients were subjected to routine intravenous sampling after fasting for 8 h on the day of admission, including urea, total protein (TP), activated partial thromboplastin time (APTT), thrombin time (TT), prothrombin (PT), calcium, triglyceride (TG), albumin (Alb), globulin (Glo), phosphorus, and D-dimer. Blood samples for testing APTT, TT, and PT were collected in vacuum blood collection tubes, and the remaining blood samples were collected with citrate anticoagulant tubes. Measurements of all biochemical parameters were performed by the Clinical Laboratory at the First Hospital of China Medical University (Shenyang, China). The aforementioned factors were taken into account as confounders in our study. We further selected potential confounders based on their association with the LOS (Supplementary Table 1) or if they changed the estimates of fibrinogen level on LOS by more than 10% (Supplementary Table 2). Moreover, we included covariates clinically relevant to LEAD and LOS. The following covariates were finally identified: age, gender, number of historical hospitalizations, surgical history, vascular disease history, drinking history, smoking history, insurance type, surgical approach, lesion site, weight loss (loss of ≥ 2 kg), Fontaine classification^20^, age-adjusted Charlson comorbidity index (ACCI)^21^, urea, TP, APTT, TT, prothrombin time-international normalized ratio (PT-INR), calcium, TG, Alb/Glo ratio, phosphorus, and D-dimer.

### Statistical analysis

#### Descriptive analysis

The Kolmogorov–Smirnov test was used to test the normality of continuous variables. All continuous variables were nonnormally distributed and are expressed as the median (interquartile range). Categorical variables were reported as numbers (percentages).

#### Variable selection

The univariate analysis was conducted to assess the association between covariates and LOS. We screened for covariates by introducing covariates in the basic model and excluding covariates in the full model, observing the change in the regression coefficient of the fibrinogen level. We also calculated variance inflation factors for each variable in generalized linear models to assess collinearity. Multicollinearity was not observed among the variables, with variance inflation coefficients of all variables less than 2.8.

#### Regression modeling

We conducted generalized linear models with a negative binomial link function to explore the association between fibrinogen level and LOS, including the crude model (controlled for no confounders), Model 2 (controlled for age and gender), and Model 3 (controlled for all confounders). Unordered categorical variables were included in the model using dummy variables. To address the skewness of LOS data, we employed the negative binomial link function within the framework of a generalized linear model. The negative binomial link function transforms the linear combination of predictor variables into non-negative, discrete count values. It accommodates situations where the mean and variance of the count data are unequal. The inclusion of a dispersion parameter allows for controlling the relationship between the variance and the mean. We also used a generalized additive model with a smoothing spline to determine the association between fibrinogen level and LOS after adjusting for possible confounders. We imputed missing baseline confounding data using multiple imputations to evaluate whether the use of indicator variables for missing data would lead to biases in our results. In addition, stratified and interaction analyses were performed based on age, gender, surgical history, vascular disease history, drinking history, smoking history, insurance type, surgical approach, lesion site, weight loss, and Fontaine classification. We also calculated the E-value to evaluate the possibility of unmeasured confounding factors between fibrinogen level and LOS. Then, we explored the correlation of fibrinogen with D-dimer, TG, ACCI, APTT, TT, and PT-INR.

#### Model assessment and validation

To assess the accuracy of fibrinogen level for predicting LOS of > 10 days (median LOS), receiver operating characteristic (ROC) curves were constructed and the areas under the curve (AUC) were compared with and without fibrinogen level. We also computed the 95% confidence interval (CI) of the AUC with and without the fibrinogen level by using the bootstrap method with 500 bootstrap samples. The calibration plot was generated by performing 500 bootstrap runs to assess model calibration, and decision curve analysis was performed to assess additional benefits.

Two-tailed *p*-values < 0.05 were considered statistically significant for all analyses. The statistical analysis was conducted using Stata version 16.0 (StataCorp, College Station, TX, USA) and R version 3.6.0 (R Foundation for Statistical Computing, Vienna, Austria).

### Ethics approval and consent to participate

The Ethics Committee of the First Hospital of China Medical University approved the study with the ethics number [2021]366. As the patient information was anonymized and deidentified before analysis, informed consent was not needed.

## Results

### Baseline characteristics

The analyzed population was comprised of 1153 males (80.74%) and 275 females, with a median age of 70 years old. A total of 1161 patients had a history of vascular disease, and 681 had a history of surgery. Table 1 summarizes the baseline clinical characteristics of the participants in this study.

**Table 1:** Baseline characteristics of the patients (n = 1428).

Variables	Value
Gender, n (%)	
Male	1153 (80.74)
Female	275 (19.26)
Age (years), median (IQR)	70.00 (64.00–77.25)
Number of historical hospitalizations (times), median (IQR)	1.00 (1.00–2.00)
Surgery history, n (%)	
Yes	681 (47.82)
No	743 (52.18)
Vascular disease history, n (%)	
Yes	1161 (81.53)
No	263 (18.47)
Drinking history, n (%)	
Yes	383 (26.90)
No	1041 (73.10)
Smoking history, n (%)	
Yes	722 (50.70)
No	702 (49.30)
Insurance type, n (%)	
Out-of-pocket	92 (6.44)
Urban and rural resident medical insurance	293 (20.52)
Social insurance	628 (43.98)
Employee medical insurance	415 (29.06)
Surgical approach, n (%)	
No surgery	330 (23.11)
Open surgery	220 (15.41)
Interventional surgery	878 (61.48)
Lesion site, n (%)	
Unilateral lower limb	676 (47.81)
Bilateral lower limbs	738 (52.19)
Weight loss, n (%)	
Yes	13 (1.48)
No	863 (98.52)
Fontaine classification, n (%)	
Class I	58 (4.37)
Class II	1078 (81.17)
Class III	33 (2.48)
Class IV	159 (11.97)
ACCI (score), median (IQR)	4.00 (3.00–5.00)
Urea (mmol/L), median (IQR)	6.16 (4.83–7.70)
PT (g/L), median (IQR)	62.70 (58.90–66.55)
APTT (s), median (IQR)	39.10 (35.80–43.20)
TT (s), median (IQR)	16.70 (16.00–17.60)
PT-INR, median (IQR)	1.02 (1.00–1.09)
Calcium (mmol/L), median (IQR)	2.19 (2.10–2.27)
TG (mmol/L), median (IQR)	1.36 (0.97–1.97)
Alb/Glo ratio	1.50 (1.20–1.70)
Phosphorus (mmol/L), median (IQR)	1.11 (0.98–1.24)
D-dimer (mg/L), median (IQR)	0.67 (0.39–1.29)
Fibrinogen (g/L), median (IQR)	3.97 (3.24–5.22)
Length of stay (day), median (IQR)	10.00 (7.00–15.00)

*IQR,* interquartile range; *ACCI*, age-adjusted Charlson comorbidity index; *TP*, total protein; *APTT*, activated partial thromboplastin time; *TT*, thrombin time; *PT-INR*, prothrombin time international normalized ratio; *TG*, triglyceride; *Alb/Glo ratio*, albumin/globulin ratio.

^a^Data presented as the median (interquartile range) for continuous variables and frequency (%) for categorical variables. Total percentages within categories may not equal 100% due to rounding.

### Association between fibrinogen level and LOS

**Figure 2:**
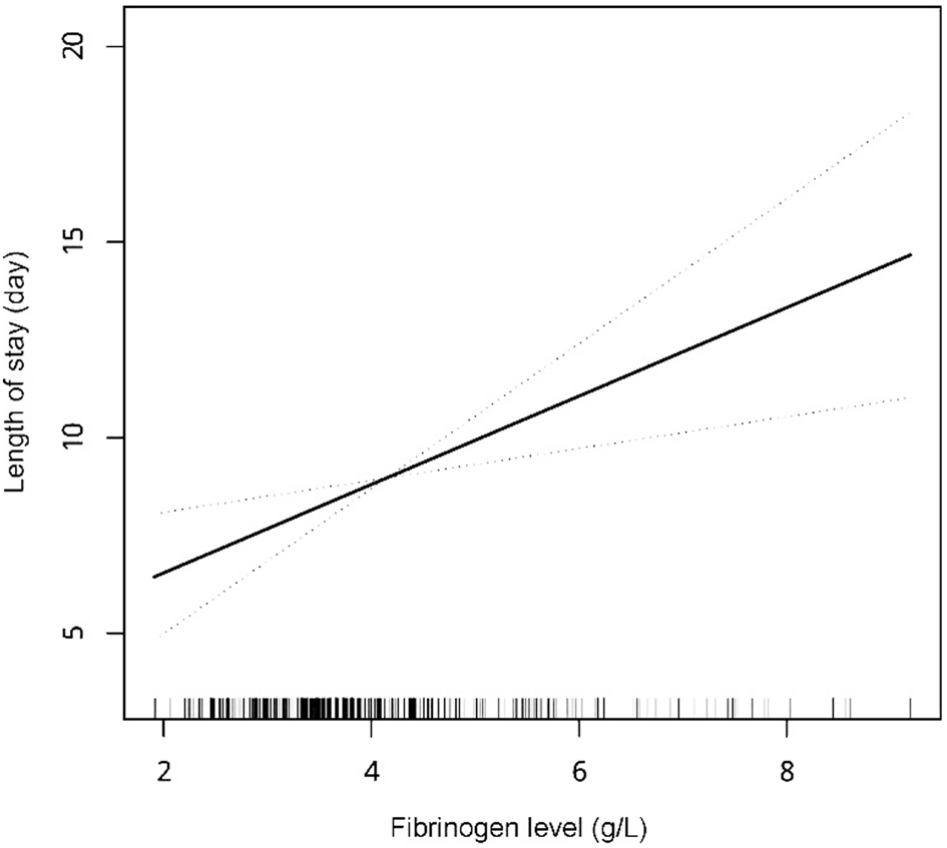
Association between fibrinogen level and length of stay.

Three models were conducted to analyze the association between fibrinogen level and LOS, as shown in Table 2. The unadjusted model showed that fibrinogen level was associated with LOS (β = 0.75; 95% CI, 0.44–1.06; *p* < 0.001). After all covariates were adjusted, the coefficient of outcome increased and remained statistically significant (β = 1.14; 95% CI, 0.42–1.86; *p* = 0.002). A positive linear relationship between fibrinogen level and LOS was also found after adjusting for all confounding factors (Fig. 2). After conducting multiple imputations of the missing baseline data (missing completely at random), the results remained robust (all* p* < 0.05) (Table 2). Additionally, the E-value was used to verify any potential unadjusted confounding, and the value was 3.90.

**Table 2:** Association of fibrinogen level with length of stay before and after performing multiple imputations (n = 1428).

Item	Model 1		Model 2		Model 3	
	Unadjusted *β* (95%CI)	*P*	Adjusted *β* (95%CI)	*p*	Adjusted *β* (95%CI)	*p*
Before performing multiple imputation						
Fibrinogen (g/L)	0.75 (0.44–1.06)	< 0.001	0.74 (0.43–1.05)	< 0.001	1.14 (0.42–1.86)	0.002
After performing multiple imputation						
Fibrinogen (g/L)	0.75 (0.44–1.06)	< 0.001	0.74 (0.43–1.05)	< 0.001	0.47 (0.13–0.81)	0.007

^a^Model 2: adjusted for age and gender.

^b^Model 3: adjusted for age, gender, number of historical hospitalizations, surgical history, vascular disease history, drinking history, smoking history, insurance type, surgical approach, lesion site, weight loss, Fontaine classification, ACCI, Urea, TP, APTT, TT, PT-INR, calcium, TG, Alb/Glo ratio, phosphorus, D-dimer.

*CI* confidence interval,* β* regression coefficients, *ACCI* age-adjusted Charlson comorbidity index, *TP* total protein, *APTT* activated partial thromboplastin time, *TT* thrombin time, *PT-INR* prothrombin time international normalized ratio, *TG* triglyceride, *Alb/Glo ratio* albumin/globulin ratio.

**Figure 3:**
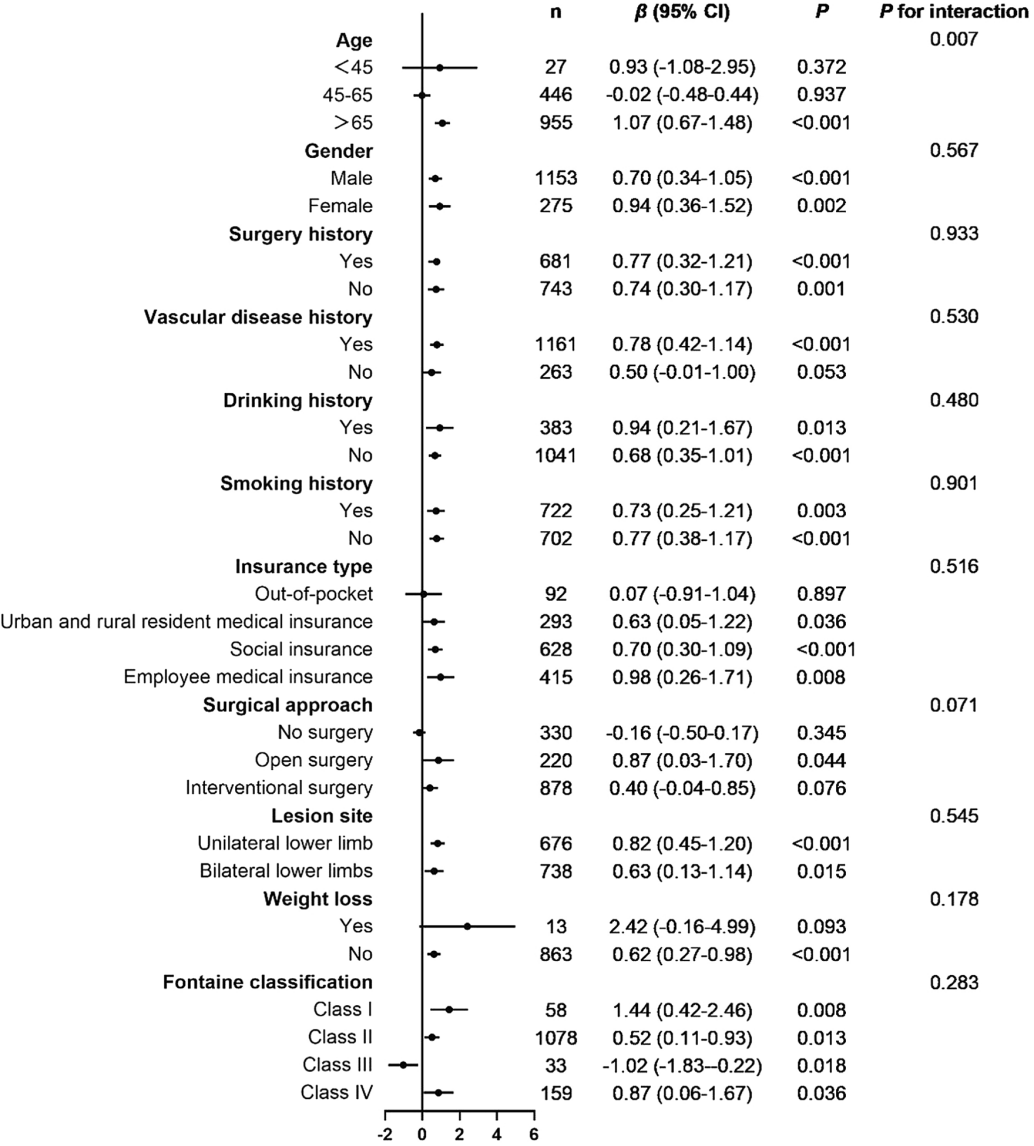
Subgroup analysis for the association between fibrinogen level and length of stay.

In different subgroups, stratified analyses were performed to investigate the association between fibrinogen level and LOS (Fig. 3). In the interaction analyses, there was no difference between fibrinogen level and LOS for most variables (all *p* for interaction > 0.05), except for age (*p* for interaction = 0.007).

Correlation analysis showed a significant relationship between fibrinogen and D-dimer, TG, ACCI, APTT, TT, and PT-INR (all *p* < 0.05) (Supplementary Fig. 1).

A comparison of the performance of discrimination for LOS of > 10 days prediction after adding fibrinogen level into the reference model is shown in Supplementary Fig. 2. The reference model included the following variables: age, gender, number of historical hospitalizations, surgical history, vascular disease history, drinking history, smoking history, insurance type, surgical approach, lesion site, weight loss, Fontaine classification, ACCI, urea, TP, APTT, TT, PT-INR, calcium, TG, Alb/Glo ratio, phosphorus, and d-dimer. In comparison to the reference model (AUC, 0.800), the fibrinogen-added model’s (AUC, 0.807) prediction accuracy improved (*p* values for the comparison > 0.05). Bootstrap analysis indicated similar results (Supplementary Table 3). The observed and predicted probabilities showed good agreement in the calibration plots, and adding a fibrinogen level improved the agreement slightly. An analysis of the decision curve indicated the potential value of the model in clinical practice (Supplementary Fig. 3).

## Discussion

A positive linear association was found between fibrinogen level and LOS in patients with LEAD. To the best of our knowledge, this study is the first to provide clear evidence of the association between fibrinogen level and LOS in LEAD patients.

Elevation of fibrinogen levels largely reflects the severity of the underlying LEAD^22^. A soluble glycoprotein in plasma, fibrinogen is involved in blood clotting response to tissue injury. After vascular injury, fibrinogen is broken down by thrombin to form fibrin, which is a major component of blood clots^23^. Fibrinogen plays several other functions in addition to its role in thrombosis, making it biologically plausible as a player in atherothrombosis. Fibrinogen can stimulate smooth muscle cells to proliferate and migrate^24^, as well as increase the levels of proinflammatory cytokines^25^. Additionally, it affects endothelial integrity and permeability of vascular walls, lures leukocytes from blood into vessels through ligand receptors, induces aggregation, and activates platelets^26,27^. The fibrin in atherosclerotic lesions is responsible for the formation of the lipid core by binding low-density lipoproteins and lipids^28^. The aforementioned mechanisms could account for the fact that elevated fibrinogen level provides useful information for estimating the likelihood across LEAD severity and likely explains the association between fibrinogen level and prolonged LOS.

In our study, correlations were observed among fibrinogen and D-dimer, ACCI, APTT, PT-INR, TG, and TT, indicating that the above factors may have synergistic effects on the severity and progression of LEAD. As a marker of hypercoagulability, D-dimer is associated with both arterial and venous thrombosis thrombotic events^29^. Dyslipidemia can increase the risk of PAD. Triglycerides were associated with incident PAD independently^30^. As a modified form of the Charlson comorbidity index, the AACI score includes age as an additional comorbidity factor^21^. A study indicated that age is one of the leading factors underlying the development and progression of both symptomatic and asymptomatic PAD^31^. Furthermore, patients with LEAD often suffer from comorbid diseases, such as heart failure, diabetes, coronary heart disease, and hypertension^32^. Age and comorbidities are factors contributing to longer LOS. Although many of these conditions have similar etiological factors, some LEAD comorbidities may arise from systemic inflammatory responses in individuals. Fibrinogen, as an inflammatory marker, might act as an indicator of the aforementioned comorbidities in LEAD patients. Fibrinogen and APTT, TT, and PT-INR could together reflect the coagulation status of patients^33^. The aforementioned findings provide valuable information on fibrinogen levels and may prolong LOS in patients with LEAD.

Despite several important findings, our study has some limitations. First, this was a single-center study, our findings were only verified in the Chinese population and treatment environment, and the verification group needs to be further expanded. And as most of the patients in our study sample were males and claudicants, the findings are only applicable to a limited group of people. Second, the occurrence of LEAD is a gradual process. This study only considered the first fibrinogen level at admission. It is unclear whether the observed effects were transient or persistent, and a longitudinal study in the same cases should be conducted in future research to confirm our results. Third, the E-value of 3.90 suggests that unmeasured confounding is unlikely to fully account for our findings. However, we could not examine all the unmeasured factors, such as physical activity and dietary factors. Finally, this study utilized a retrospective design, future prospective studies should be conducted to analyze the value of a panel of biochemical and hematological inflammatory markers, applicable to both inpatient and outpatient settings.

## Conclusions

In summary, our study results indicate that the fibrinogen level is significantly associated with a prolonged LOS in the LEAD inpatient setting. As a widely available and easily measured indicator of patients associated with a prolonged LOS, fibrinogen level may represent an attractive indicator for doctors, nurses, and hospital administrators to identify LEAD patients who will likely have a prolonged LOS.

## Supplementary Information

Supplementary Information.

## Data Availability

The datasets used and/or analyzed during the current study are available from the corresponding author upon reasonable request.
