# Supplementary material for: Association between fibrinogen level and length of stay in patients with lower extremity atherosclerotic disease: a retrospective cohort study
Source: Sci Rep. 2023 Jul 22;13:11872. doi: 10.1038/s41598-023-39219-x (PMC10363167; doi:10.1038/s41598-023-39219-x)
Supplement: Supplementary file 1 — Supplementary Information. [file 41598_2023_39219_MOESM1_ESM.pdf]

Supplementary Table 1, online only. Associations of covariates with length of stay (n = 1428)

Variable	*β* (95% CI)	*p*
Gender		
Male	Reference	-
Female	-1.75 (-3.06--0.45)	0.008
Age, years	-0.02 (-0.07-0.03)	0.425
Number of historical hospitalizations, times	0.34 (-0.01-0.69)	0.059
Surgery history		
Yes	Reference	-
No	-0.34 (-1.37-0.70)	0.520
Vascular disease history		
Yes	Reference	-
No	-1.03 (-2.36-0.30)	0.130
Drinking history		
Yes	Reference	-
No	-0.38 (-1.55-0.78)	0.519
Smoking history		
Yes	Reference	-
No	-0.70 (-1.74-0.33)	0.183
Insurance type		
Out-of-pocket	Reference	-
Urban and rural resident medical insurance	-0.24 (-2.56-2.09)	0.843
Social insurance	-0.03 (-2.20-2.15)	0.980
Employee medical insurance	0.60 (-1.64-2.85)	0.600
Surgical approach		
No surgery	Reference	-
Open surgery	9.59 (7.98-11.21)	<0.001
Interventional surgery	2.26 (1.06-3.45)	<0.001
Lesion site		
Unilateral lower limb	Reference	-
Bilateral lower limbs	-0.24 (-1.28-0.80)	0.654
Weight loss		
Yes	Reference	-
No	-2.07 (-6.60-2.46)	0.370
Fontaine classification		
Class I	Reference	-
Class II	0.92 (-1.64-3.49)	0.481
Class III	-2.46 (-6.61-1.69)	0.245
Class IV	4.27 (1.35-7.19)	0.004
ACCI, score	0.11 (-0.21-0.44)	0.499
Urea, mmol/L	-0.11 (-0.25-0.04)	0.140
TP, g/L	-0.03 (-0.11, 0.06)	0.529
APTT, s	0.03 (-0.02-0.08)	0.182
TT, s	0.01 (-0.04-0.05)	0.834
PT-INR	2.16 (-0.23-4.56)	0.077
Calcium, mmol/L	-0.27 (-3.93-3.38)	0.884
TG, mmol/L	-0.06 (-0.42-0.31)	0.766
Alb/Glo ratio	-1.72 (-4.49-1.05)	0.225
Phosphorus, mmol/L	-1.19 (-3.07-0.70)	0.219
D-dimer, mg/L	0.27 (-0.03-0.57)	0.078

CI, Confidence interval; *β,* regression coefficients; *ACCI*, age-adjusted Charlson comorbidity index; *TP*, total protein; *APTT*, activated partial thromboplastin time; *TT*, thrombin time; *PT-INR*, prothrombin time international normalized ratio; *TG*, triglyceride; *Alb/Glo ratio*, albumin/globulin ratio.

Supplementary Table 2, online only. Changes in the regression coefficients of fibrinogen level based on the introduction of covariates in the basic model and the removal of covariates in the full model

Covariate	Basic model	Full model	Selected
	0.283	1.134	
Gender	0.274	1.130	
Age, years	0.284	1.167	
Number of historical hospitalizations, times	0.281	1.128	
Surgery history	0.279	1.136	
Vascular disease history	0.268	1.145	
Drinking history	0.309	1.153	
Smoking history	0.350*	1.128	Yes
Insurance type	0.335*	1.108	Yes
Surgical approach	0.242*	1.018*	Yes
Lesion site	0.284	1.136	
Weight loss	0.282	1.139	
Fontaine classification	0.361*	1.127	Yes
ACCI, score	0.351*	1.143	Yes
Urea, mmol/L	0.347*	1.126	Yes
TP, g/L	0.257	1.110	
APTT, s	0.382*	0.904*	Yes
TT, s	0.303	0.841*	Yes
PT-INR	0.290	1.121	
Calcium, mmol/L	0.326*	1.130	Yes
TG, mmol/L	0.293	1.129	
Alb/Glo ratio	0.727*	0.653*	Yes
Phosphorus, mmol/L	0.292	1.135	
D-dimer, mg/L	0.281	1.134	

[a] * Indicated a change of more than 10% from the starting regression coefficient.

ACCI, age-adjusted Charlson comorbidity index; *TP*, total protein; *APTT*, activated partial thromboplastin time; *TT*, thrombin time; *PT-INR*, prothrombin time international normalized ratio; *TG*, triglyceride; *Alb/Glo ratio*, albumin/globulin ratio.

Supplementary Table 3, online only. Receiver operating characteristics analysis for predicting a length of stay of > 10 days

Variable	AUC (95% CI)	Optimal threshold value	Sensitivity	NPV	Specificity	PPV	*P*
**Original series**							
Reference model	0.800 (0.739-0.860)	-1.005	0.643	0.884	0.826	0.529	<0.001
Reference model + fibrinogen	0.807 (0.749-0.866)	-1.732	0.886	0.943	0.574	0.388	<0.001
**Bootstrap series**							
Reference model	0.799 (0.733-0.848)	-1.005	0.643	0.884	0.826	0.529	<0.001
Reference model + fibrinogen	0.811 (0.753-0.862)	-1.732	0.886	0.943	0.574	0.388	<0.001

[a] The reference model included age, gender, number of historical hospitalizations, surgical history, vascular disease history, drinking history, smoking history, insurance type, surgical approach, lesion site, weight loss, Fontaine classification, ACCI, urea, TP, APTT, TT, PT-INR, calcium, TG, Alb/Glo ratio, phosphorus, D-dimer.

[b] The optimal threshold is the largest cutoff value with the sensitivity + specificity.

AUC, area under the curve; *CI*, Confidence interval; *NPV*, negative predictive value; *PPV*, positive predictive value; *ACCI*, age-adjusted Charlson comorbidity index; *TP*, total protein; *APTT*, activated partial thromboplastin time; *TT*, thrombin time; *PT-INR*, prothrombin time international normalized ratio; *TG*, triglyceride; *Alb/Glo ratio*, albumin/globulin ratio.

Supplementary Figures:

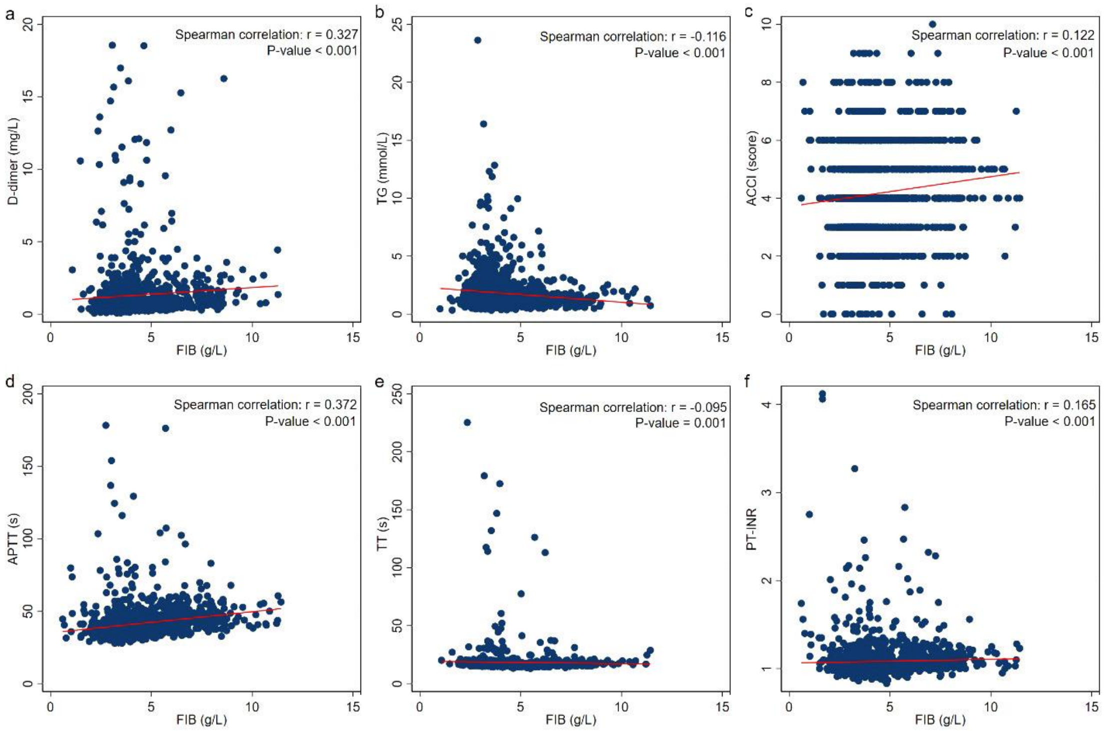

Supplementary Figure 1, online only. Correlation plots among fibrinogen and D-dimer, triglyceride, age-adjusted Charlson comorbidity index, activated partial thromboplastin time, thrombin time, and prothrombin time international normalized ratio.

FIB, fibrinogen; *TG*, triglyceride; *ACCI*, age-adjusted Charlson comorbidity index; *APTT*, activated partial thromboplastin time; *TT*, thrombin time; *PT-INR*, prothrombin time international normalized ratio.

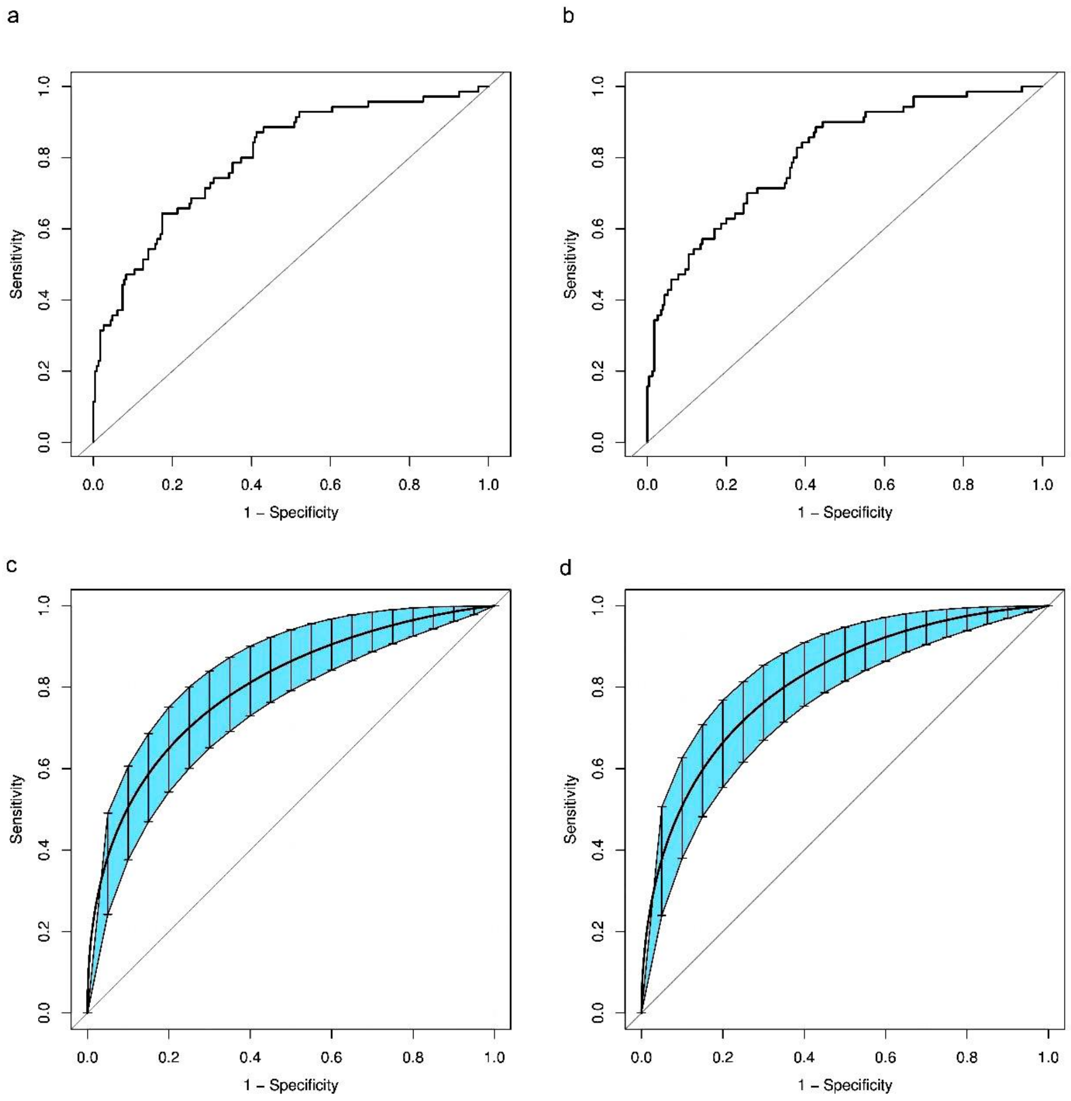

Supplementary Figure 2, online only. Receiver operating characteristic curves for predicting a length of stay of > 10 days in lower extremity atherosclerotic disease patients. **(a)** Reference model in predicting a LOS of > 10 days; **(b)** Reference model adding fibrinogen level in predicting a LOS of > 10 days; **(c)** Reference model in predicting a LOS of > 10 days by using bootstrap resampling (500 times); **(d)** Reference model adding fibrinogen level in predicting a LOS of > 10 days by using bootstrap resampling (500 times). The reference model included age, gender, number of historical hospitalizations, surgical history, vascular disease history, drinking history, smoking history, insurance type, surgical approach, lesion site, weight loss, Fontaine classification, ACCI, urea, TP, APTT, TT, PT-INR, calcium, TG, Alb/Glo ratio,

phosphorus, D-dimer.

LEAD, Lower extremity arterial disease; *LOS*, length of stay; *ACCI*, age-adjusted Charlson comorbidity index; *TP*, total protein; *APTT*, activated partial thromboplastin time; *TT*, thrombin time; *PT-INR*, prothrombin time international normalized ratio; *TG*, triglyceride; *Alb/Glo ratio*, albumin/globulin ratio.

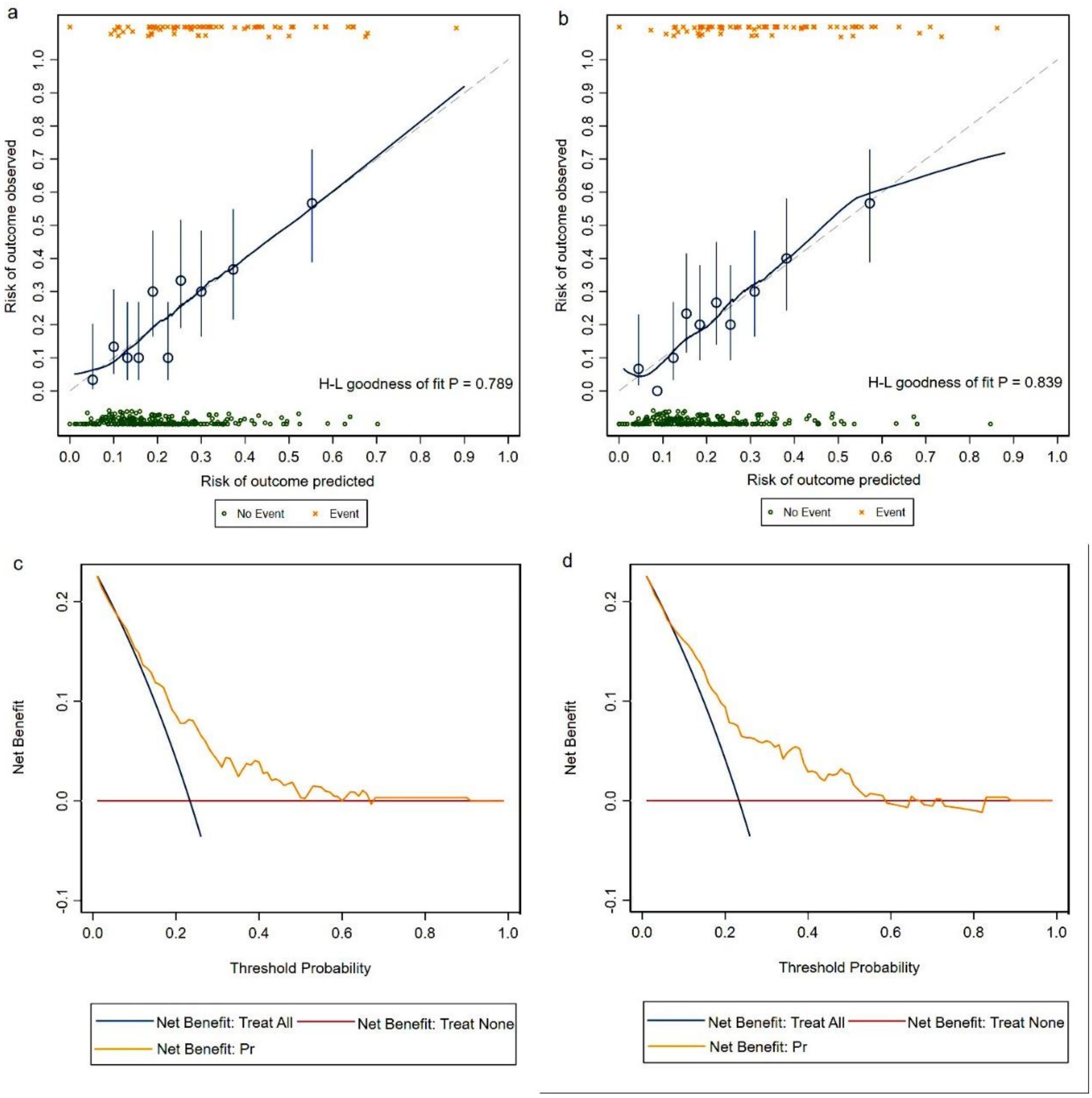

Supplementary Figure 3, online only. Calibration plots and decision curve analysis for predicting a length of stay of > 10 days in lower extremity atherosclerotic disease patients. **(a)** Calibration plot: reference model in predicting a LOS of > 10 days; **(b)** Calibration plot: reference model adding fibrinogen level in predicting a LOS of > 10 days. Prediction accuracy increased as the solid line approached the dotted line. **(c)**

Decision curve analysis: reference model in predicting a LOS of > 10 days; **(d)** Decision curve analysis: reference model adding fibrinogen level in predicting a LOS of > 10 days. Solid orange line is from prediction model, blue line is for all patients with LOS of > 10 days, and the horizontal red line indicates no patients with LOS of > 10 days. The reference model included age, gender, number of historical hospitalizations, surgical history, vascular disease history, drinking history, smoking history, insurance type, surgical approach, lesion site, weight loss, Fontaine classification, ACCI, urea, TP, APTT, TT, PT-INR, calcium, TG, Alb/Glo ratio, phosphorus, D-dimer.

LEAD, Lower extremity arterial disease; *LOS*, length of stay; *ACCI*, age-adjusted Charlson comorbidity index; *TP*, total protein; *APTT*, activated partial thromboplastin time; *TT*, thrombin time; *PT-INR*, prothrombin time international normalized ratio; *TG*, triglyceride; *Alb/Glo ratio*, albumin/globulin ratio.
